# Are oval fat bodies useful diagnostic clues in kidney disease?

**DOI:** 10.1093/ckj/sfaf281

**Published:** 2025-09-13

**Authors:** Eisuke Kubo, Kotaro Haruhara, Takaya Sasaki, Nobuo Tsuboi, Takashi Yokoo

**Affiliations:** Division of Nephrology and Hypertension, Department of Internal Medicine, Jikei University School of Medicine, Tokyo, Japan; Division of Nephrology and Hypertension, Department of Internal Medicine, Jikei University School of Medicine, Tokyo, Japan; Division of Nephrology and Hypertension, Department of Internal Medicine, Jikei University School of Medicine, Tokyo, Japan; Division of Nephrology and Hypertension, Department of Internal Medicine, Jikei University School of Medicine, Tokyo, Japan; Division of Nephrology and Hypertension, Department of Internal Medicine, Jikei University School of Medicine, Tokyo, Japan

To the Editor,

Oval fat bodies (OFBs) are a characteristic finding in urinary sediments and are considered a hallmark of nephrotic syndrome [1]. They appear as a distinctive Maltese cross pattern when viewed under polarized light microscopy and represent swollen tubular epithelial cells filled with reabsorbed lipoproteins [2, 3]. The clinical use of OFBs has largely been confined to their role in confirming the presence of nephrotic syndrome, with little attention paid to their potential in distinguishing between underlying glomerular diseases.

We conducted a cross-sectional study to assess the diagnostic relevance of urinary OFBs in patients undergoing native kidney biopsy at our institution between 2011 and 2017. This study was approved by the Institutional Review Board of the Jikei University School of Medicine [no. 34-104 (11251)]. The patient demographics and clinical characteristics are shown in Supplementary Table S1.

The study population included 676 consecutive patients and 177 patients (26%) had urinary OFBs. Notably, the presence of OFBs was significantly associated with a lower estimated glomerular filtration rate, higher urinary protein excretion and a higher prevalence of nephrotic syndrome. OFB positivity across disease aetiologies is shown in Fig. 1. Histopathological diagnoses most frequently associated with OFBs include membranous nephropathy and membranoproliferative glomerulonephritis, both of which are classically associated with nephrotic syndrome and marked glomerular injury.

**Figure 1:**
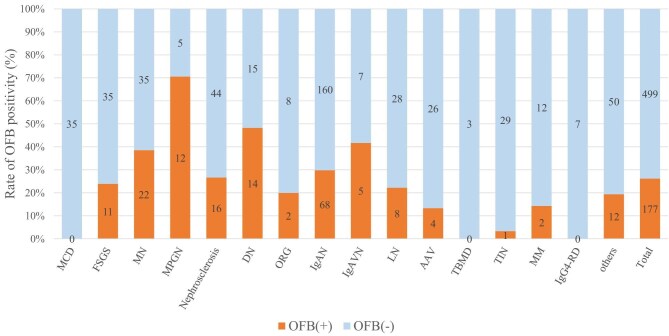
The proportion of OFB positivity across different kidney disease aetiologies. This graph presents the proportion of OFB positivity in patients who underwent native kidney biopsies for various kidney diseases. Orange bars indicate OFB-positive cases, whereas light blue bars represent OFB-negative cases. The numbers on the bars denote the number of patients with each aetiology. AAV: anti-neutrophil cytoplasmic antibody-associated vasculitis; DN: diabetic nephropathy; FSGS: focal segmental glomerulosclerosis; IgAN: IgA nephropathy; IgAVN: IgA vasculitis with nephritis; LN: lupus nephritis; IgG4-RD: IgG4-related disease; MM: multiple myeloma; MN: membranous nephropathy; MPGN: membranoproliferative glomerulonephritis; ORG: obesity-related glomerulopathy; TBMD: thin basement membrane disease; TIN: tubulointerstitial nephritis.

Our data suggest that OFBs are not strictly confined to nephrotic syndrome patients. Several patients with subnephrotic proteinuria also demonstrate OFBs in their urinary sediments. Conditions such as focal segmental glomerulosclerosis and lupus nephritis, both characterized by podocyte injury, exhibit variable OFB positivity. This finding implies that OFBs may reflect ongoing podocyte injury and abnormal tubular protein handling rather than merely the degree of proteinuria. In contrast, OFBs are rarely detected in diseases with predominantly tubulointerstitial pathology, such as tubulointerstitial nephritis. This relative absence supports the notion that OFBs are more specific for glomerular injury and not merely by-products of generic urinary protein leakage.

Strikingly, no patient with minimal change disease (MCD), a leading cause of nephrotic syndrome, exhibited OFBs in their urinary sediments. This finding suggests that the presence of OFBs in a patient with nephrotic syndrome has high specificity for ruling out MCD, thereby shifting the diagnostic consideration toward other glomerulopathies. Kidney biopsy remains the gold standard for diagnosing MCD; however, light microscopy findings are often normal and the diagnosis relies on the exclusion of other glomerulopathies [4]. Given that renal biopsies are invasive and subject to sampling error, especially in diseases with subtle or focal histological changes, assessment of OFBs could serve as a simple, non-invasive adjunct that helps narrow the differential diagnosis, particularly in cases with equivocal biopsy findings.

Taken together, these findings prompted a re-evaluation of OFBs in nephrology. Although their association with nephrotic syndrome is well accepted, our study highlights the nuanced patterns of their presence and absence across distinct glomerular pathologies. Rather than being viewed as mere epiphenomena, OFBs may offer clinically relevant information that complements the histological diagnosis, especially in scenarios of limited biopsy yield or when a biopsy is contraindicated.

In conclusion, urinary OFBs represent a potentially valuable and underutilized tool in the diagnostic workup of glomerular diseases. Their presence may indicate a glomerular origin and severity of injury. In patients with nephrotic syndrome, their presence may help argue against MCD. Further prospective studies are needed to validate these observations and establish standardized criteria for the assessment of OFBs in clinical practice. However, our study has some limitations. Namely, it was conducted at a single centre, it relied on retrospective data collection and it employed a cross-sectional design, which does not allow us to address any causal relationships. Nonetheless, our findings suggest that careful examination of urinary sediment remains a vital and informative component of nephrological evaluation.

## Supplementary Material

sfaf281_Supplemental_File

## Data Availability

The data supporting this study's findings are available from the corresponding authors upon request. However, because of privacy or ethical restrictions, they are not publicly available.
