# Supplementary material for: Are oval fat bodies useful diagnostic clues in kidney disease?
Source: Clin Kidney J. 2025 Sep 13;18(10):sfaf281. doi: 10.1093/ckj/sfaf281 (PMC12498091; doi:10.1093/ckj/sfaf281)
Supplement: sfaf281_Supplemental_File [file sfaf281_supplemental_file.docx]

**Supplementary Material**

**Are Oval Fat Bodies Useful Diagnostic Clues in Kidney Disease?**

Eisuke Kubo, Kotaro Haruhara, Takaya Sasaki, Nobuo Tsuboi, Takashi Yokoo

Division of Nephrology and Hypertension Department of Internal Medicine, Jikei University School of Medicine, Tokyo, Japan

**Supplementary Materials**

**Supplementary Table S1. Clinical characteristics of patients with or without oval fat bodies (OFBs).**

|  | OFB– (n = 499) | OFB+ (n = 177) | p value |
| --- | --- | --- | --- |
| Male Sex, n (%) | 286 (57) | 130 (73) | <0.001 |
| Age, years | 51.3 ± 16.9 | 52.6 ± 16.9 | 0.393 |
| BMI, kg/m^2^ | 23.1 ± 4.3 | 24.8 ± 4.4 | <0.001 |
| eGFR, mL/min/1.73m^2^ | 56.6 ± 29.4 | 47.4 ± 25.2 | <0.001 |
| UPE, g/day | 0.87 [0.40, 2.20] | 3.76 [1.71, 5.34] | <0.001 |
| NS, n (%) | 70 (14) | 69 (39) | <0.001 |
| LDL-C, mg/dL | 146.8 ± 77.1 | 164.0 ± 89.3 | 0.082 |
| HDL-C, mg/dL | 62.7 ± 23.4 | 60.1 ± 20.2 | 0.373 |
| TG, mg/dL | 161.1 ± 100.5 | 185.87 ± 117.9 | 0.046 |

Values are presented as the mean (standard deviation) for normally distributed data and as the median [interquartile range] for non-normally distributed data. Statistical comparisons were performed using Fisher's exact test for categorical variables, Welch's *t-*test for normally distributed continuous variables, and the Mann-Whitney U test for non-normally distributed continuous variables.

Abbreviations: BMI, body mass index; eGFR, estimated glomerular filtration rate; HDL-C, high-density lipoprotein cholesterol; LDL-C, low-density lipoprotein cholesterol; NS, nephrotic syndrome; OFB, oval fat bodies; TG, triglyceride; UPE, urinary protein excretion.
